# Supplementary material for: Deciphering caveolar functions by syndapin III KO-mediated impairment of caveolar invagination
Source: eLife. 2017 Dec 5;6:e29854. doi: 10.7554/eLife.29854 (PMC5716666; doi:10.7554/eLife.29854)
Supplement: Transparent reporting form [file elife-29854-transrepform.docx]

***eLife’s* transparent reporting form**

We encourage authors to provide detailed information *within their submission* to facilitate the interpretation and replication of experiments. Authors can upload supporting documentation to indicate the use of appropriate reporting guidelines for health-related research (see [EQUATOR Network](http://www.equator-network.org/%20)), life science research (see the [BioSharing Information Resource](https://biosharing.org/)), or the [ARRIVE guidelines](http://www.plosbiology.org/article/info:doi/10.1371/journal.pbio.1000412) for reporting work involving animal research. Where applicable, authors should refer to any relevant reporting standards documents in this form.

If you have any questions, please consult our Journal Policies and/or contact us: [editorial@elifesciences.org](mailto:editorial@elifesciences.org).

**Sample-size estimation**

- You should state whether an appropriate sample size was computed when the study was being designed
- You should state the statistical method of sample size computation and any required assumptions
- If no explicit power analysis was used, you should describe how you decided what sample (replicate) size (number) to use

Please outline where this information can be found within the submission (e.g., sections or figure legends), or explain why this information doesn’t apply to your submission:

This information does not apply. We did not conduct animal experience with the goal to compare the performance of two defined cohorts of animals.

Instead, we exclusively used animals to obtain tissue and cellular material. In this case, the biological variance can be assumed to mainly be represented in number of individual cells analyzed (biological error; requires sufficient number of cells = images analyzed) and in the preparation procedure (technical error; requires confirmation by independent experiments/preparations). Please also see below.

**Replicates**

- You should report how often each experiment was performed
- You should include a definition of biological versus technical replication
- The data obtained should be provided and sufficient information should be provided to indicate the number of independent biological and/or technical replicates
- If you encountered any outliers, you should describe how these were handled
- Criteria for exclusion/inclusion of data should be clearly stated
- High-throughput sequence data should be uploaded before submission, with a private link for reviewers provided (these are available from both GEO and ArrayExpress)

Please outline where this information can be found within the submission (e.g., sections or figure legends), or explain why this information doesn’t apply to your submission:

The material and method section states that in total, 1087 µm2 membrane were scored for quantitative freeze fracture analyses and 2889 caveolin-positive invaginations were evaluated. All other information is given in the legends of the figures.

The number of individual cells (= images) analyzed (biological error) is given in the legends of the figures.

To exclude technical errors, all quantitative data sets are underscored by verifications with independent preparations of material (their n-numbers also is reported in the legends).

Qualitative data shown is of course reproduced at least by one independent experiment. This is simply according to general good scientific practice and should of course go without saying. If eLIFE guidelines for some reasons require an explicit statement of this in any single case, we shall be happy to add. But as mentioned, should be clear anyway.

In additions, the legend usually also specify experiments that

a) were done in a blinded manner and

b) in addition often have even been conducted by an independent experimentator.

Outlying data points were not excluded from any of our quantitative analyses, as there are in our eyes no clear scientific reasons why such (increased) experimental noise should be suppressed. It is part of the biological (or technical) data variance one has to accept.

Our study does not contain any high-through put data sets.

**Statistical reporting**

- Statistical analysis methods should be described and justified
- Raw data should be presented in figures whenever informative to do so (typically when N per group is less than 10)
- For each experiment, you should identify the statistical tests used, exact values of N, definitions of center, methods of multiple test correction, and dispersion and precision measures (e.g., mean, median, SD, SEM, confidence intervals; and, for the major substantive results, a measure of effect size (e.g., Pearson's r, Cohen's d)
- Report exact p-values wherever possible alongside the summary statistics and 95% confidence intervals. These should be reported for all key questions and not only when the p-value is less than 0.05.

Please outline where this information can be found within the submission (e.g., sections or figure legends), or explain why this information doesn’t apply to your submission:

Statistical significance calculations were carried out in all quantitative evaluations and the results are marked in all figures.

Statistical tests and post-tests used are reported in the figure legends in all cases (where applicable, 2-tailed Student’s t test was used; other data sets required 1-way Anova with Tukey’s post-test or 2-way ANOVA and Bonferroni post-test). Data are usually mean±SEM. Also this is specified in all cases in the legends of the figures.

(For large datasets, or papers with a very large number of statistical tests, you may upload a single table file with tests, Ns, etc., with reference to sections in the manuscript.)

**Group allocation**

- Indicate how samples were allocated into experimental groups (in the case of clinical studies, please specify allocation to treatment method); if randomization was used, please also state if restricted randomization was applied
- Indicate if masking was used during group allocation, data collection and/or data analysis

Please outline where this information can be found within the submission (e.g., sections or figure legends), or explain why this information doesn’t apply to your submission:

Does not apply. Experimental groups were defined by genotypes determined and/or conditions tested.

A significant part of the study has been done in a blinded manner (samples encoded by numbers and scrambled by independent experimentator; evaluated; then decoded).

In addition, significant parts of the evaluations have even been experimentally conducted by independent experimentators (splits of sample recordings and evaluations).

**Additional data files (“source data”)**

- We encourage you to upload relevant additional data files, such as numerical data that are represented as a graph in a figure, or as a summary table
- Where provided, these should be in the most useful format, and they can be uploaded as “Source data” files linked to a main figure or table
- Include model definition files including the full list of parameters used
- Include code used for data analysis (e.g., R, MatLab)
- Avoid stating that data files are “available upon request”

Please indicate the figures or tables for which source data files have been provided:

We are grateful for the opportunity to provide the numerical data and/or the full raw data shown in the plethora of quantitative analyses that are part of our study. Source data files are provided for all quantitative panels in Figure 1-10 and for the Suppl. Data to Figure 6 and 8 (included in the Figure 6 and 8 source data files).
